# EORTC stomach cancer PD-L1 biomarker European initiative: the ASPIRE study protocol

**DOI:** 10.1016/j.esmogo.2024.100071

**Published:** 2024-07-10

**Authors:** A. Petrillo, L. Oudijk, R. Sundar, C. Daumer, J. Casas, D. D’Haese, M. Mauer, N. van Grieken, E.C. Smyth, M. Moehler

**Affiliations:** 1 Medical Oncology Unit, Ospedale del Mare, Naples, Italy; 2 Erasmus MC, Rotterdam, The Netherlands; 3 Yong Loo Lin School of Medicine, National University of Singapore, Singapore; 4 Department of Haematology-Oncology, National University Cancer Institute, Singapore, Singapore; 5 European Organisation for Research and Treatment of Cancer, Brussels, Belgium; 6 Amsterdam UMC, Amsterdam, The Netherlands; 7 Department of Oncology, Oxford University Hospitals NHS Foundation Trust, Oxford, UK; 8 Johannes Gutenberg University Clinic, Mainz, Germany

**Keywords:** PD-L1, combined positive score, immunotherapy, tumour area positivity

## Abstract

The evaluation of programmed death-ligand 1 (PD-L1) expression and the methodology employed are central to identify suitable candidates for immunotherapy among patients with gastro-oesophageal cancer (GC). Yet, there are no comprehensive global studies comparing the various methods and antibodies utilized for assessing PD-L1 positivity in GC. The ASPIRE study, led by the European Organisation for Research and Treatment of Cancer Gastrointestinal Tract Group (EORTC GITCG) and the National University Health System, Singapore, seeks to standardize the assessment of PD-L1 expression in GC. By comparing various PD-L1 scoring systems and assays, the study aims to simplify and harmonize the quantification and qualification of PD-L1 expression. Ultimately, this effort aims to facilitate the translation of endpoints in companion diagnostic settings. Here, we report the protocol of the study.

## Highlights

• The ASPIRE study is the first global study evaluating the interchangeability of PD-L1 scoring systems and assays in GC.
• An international panel of pathologists will analyse PD-L1 by comparing combined positive score and tumour area positivity.
• Study about the interchangeability of the PD-L1 assay clones 22C3, SP263 and 28-8.

## Graphical abstract

**Figure:**
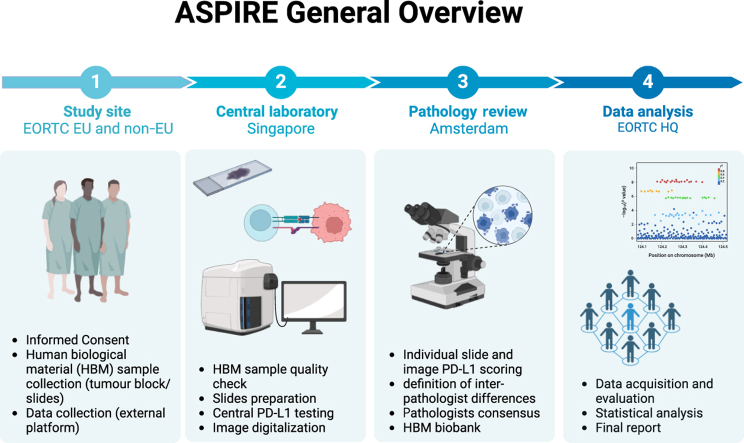

Subject area	Medicine and Dentistry
**More specific subject area**	Gastro-oesophageal adenocarcinoma
**Name of your trial in progress**	ASPIRE
**Reagents/tools**	- Dako PD-L1 clones 28-8 and 22C3 pharmDx assay (Agilent Technologies, Santa Clara, CA) - PD-L1 clone SP263 (Ventana Medical Systems Inc, Tucson, AZ)
**Trial design**	1. **Experimental description and trial design** The evaluation of programmed death-ligand 1 (PD-L1) and its methodology are central to identifying suitable candidates for immunotherapy in gastro-oesophageal cancer (GC). Yet, no studies are currently comparing PD-L1 assessment methods and immunohistochemistry (IHC) antibodies used in this context. Through this study, we seek to compare various PD-L1 scoring systems and assays, aiming to streamline and standardize the quantification and qualification of PD-L1 expression in GC. This study aims to collect ∼150 archival samples from GC through the European Organisation for Research and Treatment of Cancer (EORTC) network across Europe and with the help of international collaborators from Asia to ensure global diverse representation. Biopsy or surgical specimen formalin-fixed, paraffin-embedded blocks will be sent centrally to the laboratory at National University Health System, Singapore, where the PD-L1 staining will be carried out in a centralized and standardized manner using the PD-L1 IHC 22C3 pharmDx and PD-L1 28-8 pharmDx assays and Ventana PD-L1 (SP263). Subsequently, a group of 10-15 international gastrointestinal experts and clinically active pathologists will score the samples for PD-L1 using the combined positive score (CPS) and tumour area positivity (TAP). These scores will then be merged with clinical and pathological data. Agreement and interchangeability among the different scoring systems and assays will be assessed. 2. **Objectives and hypothesis of the project** 2.1 *Study objectives* 2.1.1 Primary objective • To measure the interchangeability of the PD-L1 expression among IHC assays 22C3, SP263 and 28-8 • To compare the scoring systems CPS and TAP in samples of GC 2.2 *Endpoints* 2.2.1 Primary endpoint • The agreement among PD-L1 expression status after dichotomizing the TAP and CPS scores at 1 and 5 cut-off 2.2.2 Secondary endpoints • The difference among the CPS and TAP scores of GC samples stained with PD-L1 22C3, SP263 and 28-8 antibodies • The concordance among PD-L1 expression status after dichotomizing the TAP and CPS scores at other cut-offs than 1 and 5 • The cut-off that would result in the smallest difference among IHC assays • The inter-pathologist variability, quantified using the intraclass correlation coefficient (ICC) of CPS and TAP scores • The inter-pathologist variability, quantified using the Fleiss kappa (κ) statistic (FKS) on dichotomized scores at multiple cut-off levels 2.2.3 Exploratory endpoints • Co-expression of PD-L1 with other biomarkers of interest including Epidermal growth factor receptor 2 (HER2), Claudin 18.2 (CLDN18.2) and Fibroblastic growth factor receptor 2b (FGFR2b) • To estimate absolute performance among the IHC assays: differences in CPS and TAP scores of positive and negative control cell lines stained with PD-L1 22C3, SP263 and 28-8 antibodies 3. **Study population** 3.1 *Population sample size* • Approximately 150 GC samples 3.2 *Patient selection criteria* • Adult (≥18 years old) • Histologically diagnosed with gastric or gastro-oesophageal junctional adenocarcinoma • Availability of tumour samples obtained within the past 3 years from biopsy or resection 4. **Research methodology and statistical plan** 4.1 *Sample size calculation*The number of GC samples needed for this study was estimated based on: • A lower boundary of 80% overall rate of agreement (ORA) below which two assays are considered significantly different • A two-sided type I error constraint alpha of 0.015 accounting for the three comparisons (among the three assays) • A two-sided type II error constraint beta of 0.20 A sample size of *n* = 127 is needed for the lower boundary of a one-sided 95% confidence interval (CI) around a true rounded-off ORA of 90% to remain above 80%. Taking a margin of 10% resulted in an estimated sample size of *n* = 140. 4.2 *Assessment of relative performance* 4.2.1 Primary endpoints The binary primary endpoint will be assessed through three 2 × 2 contingency tables depicting the number of slides that agree (*n*++ and *n*−−) or do not agree (*n*+− and *n*−+) between two candidate IHC assays. For each contingency table, the sum of all counts in the table (*n*) corresponds to the number of slides for which both assays have produced a valid PD-L1 expression status. The association between the qualitative results of two IHC assays will be presented as the ORA: $\text{ORA}=\frac{n+++n--}{n}$ The ORA between two IHC assays will always be accompanied by the 95% CI calculated by Wilson. 4.2.2 Secondary endpoints The continuous secondary endpoint on PD-L1 expression will be assessed through the within-slide differences among the mean CPS and TAP scores of the IHC assays and their statistical significance. Three assays will have three such inter-assay differences, which will be calculated. The concordance among PD-L1 expression status after dichotomizing TAP and CPS scores at other cut-offs than 1 and 5 will be calculated as explained in 4.2.1. The best cut-off, i.e. the cut-off that results in the smallest difference among IHC assays, will be established as the cut-off that maximizes the sum of the positive (PA) and negative agreement (NA), where PA and NA are the symmetric counterparts of the positive percent agreement and negative percent agreement as defined by the Food and Drug Administration. The inter-pathologist variability will be quantified by way of the ICC when dealing with continuous CPS and TAP scores and with the FKS when dealing with dichotomized PD-L1 statuses. For each combination of scoring system (TAP and CPS) and PD-L1 IHC assay (22C3, SP263 and 28-8), the continuous or dichotomized scores will be summarized allowing to assess whether the pathologist bias is comparable among scoring systems and IHC assays. The relation between the cut-off value and the amount of inter-pathologist variation will be demonstrated for the dichotomized scores. 4.2.3 Exploratory endpoints The co-expression of PD-L1 with other biomarkers of interest, including HER2, CLDN18.2 and FGFR2b, will be tabulated. Co-expression will be tested using multiple IHC with a panel of various markers, including, but not limited to, CD45, CD8, CD4, CD68, FGFR, CLDN18.2, CEACAM5, TROP2, HER2, EBER, PanCK and SMA. To estimate the feasibility to measure the absolute performance among the IHC assays: differences in CPS and TAP scores of positive and negative control cell lines stained with PD-L1 22C3, SP263 and 28-8 antibodies will also be compared. If sufficient data are available, PA and NA will be calculated and displayed alongside confusion matrices, providing information on whether the higher degree of staining of some of the IHC assays correspond to a higher PA or a lower NA. 4.3 *Clinical data collection*The following data will be collected at study entry, including but not limited to: • Demographic data: age and sex; • Basic relevant laboratory assessment, if available; • Primary disease information: date of diagnosis, primary tumour location (gastric versus gastro-oesophageal junction), histology, staging according to TNM (tumour–node–metastasis) based on American Joint Committee on Cancer latest recommendations; • Results of locally carried out tumour biomarker analyses, if available (e.g. HER2, microsatellite status, others according to local procedures, such as CLDN18.2 and FGFR2b); • Type of treatment received and setting, if available, including specifying if they included immunotherapy; and • Type of tumour specimen (from primary tumour versus metastatic sites; biopsy versus resection). Sites will be encouraged to prioritize biopsies from primary tumours as the predominant source of samples. However, samples from surgical resection sites and/or metastatic tumour biopsies will also be included to keep the study design pragmatic and real world. Minimally 30% of samples will be from primary tumour endoscopic biopsies and not more than 10% of samples from metastatic sites.
**Trial registration**	EORTC-RP-2321
**Ethics**	The study is conducted in adherence to the latest Declaration of Helsinki and Ethical Guidelines for Clinical Studies. The study protocol has received approval from the independent ethical review committee of EORTC and has been submitted to the local ethical committees. Before enrolment, written informed consent will be obtained from all patients. In specific sites, patients who have passed away may have sample access with a waiver of consent after local ethics approval.
**Value of the trial in progress**	• The ASPIRE study is the first global study evaluating the interchangeability of PD-L1 scoring systems and assays in GC. • The study will provide data to harmonize the quantification and qualification of PD-L1 expression in GC. • The results of the trial will help better select patients who derive benefit when treated with immunotherapy.

**Table:** Specifications table

Subject area	Medicine and Dentistry
**More specific subject area**	Gastro-oesophageal adenocarcinoma
**Name of your trial in progress**	ASPIRE
**Reagents/tools**	- Dako PD-L1 clones 28-8 and 22C3 pharmDx assay (Agilent Technologies, Santa Clara, CA) - PD-L1 clone SP263 (Ventana Medical Systems Inc, Tucson, AZ)
**Trial design**	1. **Experimental description and trial design** The evaluation of programmed death-ligand 1 (PD-L1) and its methodology are central to identifying suitable candidates for immunotherapy in gastro-oesophageal cancer (GC). Yet, no studies are currently comparing PD-L1 assessment methods and immunohistochemistry (IHC) antibodies used in this context. Through this study, we seek to compare various PD-L1 scoring systems and assays, aiming to streamline and standardize the quantification and qualification of PD-L1 expression in GC. This study aims to collect ∼150 archival samples from GC through the European Organisation for Research and Treatment of Cancer (EORTC) network across Europe and with the help of international collaborators from Asia to ensure global diverse representation. Biopsy or surgical specimen formalin-fixed, paraffin-embedded blocks will be sent centrally to the laboratory at National University Health System, Singapore, where the PD-L1 staining will be carried out in a centralized and standardized manner using the PD-L1 IHC 22C3 pharmDx and PD-L1 28-8 pharmDx assays and Ventana PD-L1 (SP263). Subsequently, a group of 10-15 international gastrointestinal experts and clinically active pathologists will score the samples for PD-L1 using the combined positive score (CPS) and tumour area positivity (TAP). These scores will then be merged with clinical and pathological data. Agreement and interchangeability among the different scoring systems and assays will be assessed. 2. **Objectives and hypothesis of the project** 2.1 *Study objectives* 2.1.1 Primary objective • To measure the interchangeability of the PD-L1 expression among IHC assays 22C3, SP263 and 28-8 • To compare the scoring systems CPS and TAP in samples of GC 2.2 *Endpoints* 2.2.1 Primary endpoint • The agreement among PD-L1 expression status after dichotomizing the TAP and CPS scores at 1 and 5 cut-off 2.2.2 Secondary endpoints • The difference among the CPS and TAP scores of GC samples stained with PD-L1 22C3, SP263 and 28-8 antibodies • The concordance among PD-L1 expression status after dichotomizing the TAP and CPS scores at other cut-offs than 1 and 5 • The cut-off that would result in the smallest difference among IHC assays • The inter-pathologist variability, quantified using the intraclass correlation coefficient (ICC) of CPS and TAP scores • The inter-pathologist variability, quantified using the Fleiss kappa (κ) statistic (FKS) on dichotomized scores at multiple cut-off levels 2.2.3 Exploratory endpoints • Co-expression of PD-L1 with other biomarkers of interest including Epidermal growth factor receptor 2 (HER2), Claudin 18.2 (CLDN18.2) and Fibroblastic growth factor receptor 2b (FGFR2b) • To estimate absolute performance among the IHC assays: differences in CPS and TAP scores of positive and negative control cell lines stained with PD-L1 22C3, SP263 and 28-8 antibodies 3. **Study population** 3.1 *Population sample size* • Approximately 150 GC samples 3.2 *Patient selection criteria* • Adult (≥18 years old) • Histologically diagnosed with gastric or gastro-oesophageal junctional adenocarcinoma • Availability of tumour samples obtained within the past 3 years from biopsy or resection 4. **Research methodology and statistical plan** 4.1 *Sample size calculation*The number of GC samples needed for this study was estimated based on: • A lower boundary of 80% overall rate of agreement (ORA) below which two assays are considered significantly different • A two-sided type I error constraint alpha of 0.015 accounting for the three comparisons (among the three assays) • A two-sided type II error constraint beta of 0.20 A sample size of *n* = 127 is needed for the lower boundary of a one-sided 95% confidence interval (CI) around a true rounded-off ORA of 90% to remain above 80%. Taking a margin of 10% resulted in an estimated sample size of *n* = 140. 4.2 *Assessment of relative performance* 4.2.1 Primary endpoints The binary primary endpoint will be assessed through three 2 × 2 contingency tables depicting the number of slides that agree (*n*++ and *n*−−) or do not agree (*n*+− and *n*−+) between two candidate IHC assays. For each contingency table, the sum of all counts in the table (*n*) corresponds to the number of slides for which both assays have produced a valid PD-L1 expression status. The association between the qualitative results of two IHC assays will be presented as the ORA: $\text{ORA}=\frac{n+++n--}{n}$ The ORA between two IHC assays will always be accompanied by the 95% CI calculated by Wilson. 4.2.2 Secondary endpoints The continuous secondary endpoint on PD-L1 expression will be assessed through the within-slide differences among the mean CPS and TAP scores of the IHC assays and their statistical significance. Three assays will have three such inter-assay differences, which will be calculated. The concordance among PD-L1 expression status after dichotomizing TAP and CPS scores at other cut-offs than 1 and 5 will be calculated as explained in 4.2.1. The best cut-off, i.e. the cut-off that results in the smallest difference among IHC assays, will be established as the cut-off that maximizes the sum of the positive (PA) and negative agreement (NA), where PA and NA are the symmetric counterparts of the positive percent agreement and negative percent agreement as defined by the Food and Drug Administration. The inter-pathologist variability will be quantified by way of the ICC when dealing with continuous CPS and TAP scores and with the FKS when dealing with dichotomized PD-L1 statuses. For each combination of scoring system (TAP and CPS) and PD-L1 IHC assay (22C3, SP263 and 28-8), the continuous or dichotomized scores will be summarized allowing to assess whether the pathologist bias is comparable among scoring systems and IHC assays. The relation between the cut-off value and the amount of inter-pathologist variation will be demonstrated for the dichotomized scores. 4.2.3 Exploratory endpoints The co-expression of PD-L1 with other biomarkers of interest, including HER2, CLDN18.2 and FGFR2b, will be tabulated. Co-expression will be tested using multiple IHC with a panel of various markers, including, but not limited to, CD45, CD8, CD4, CD68, FGFR, CLDN18.2, CEACAM5, TROP2, HER2, EBER, PanCK and SMA. To estimate the feasibility to measure the absolute performance among the IHC assays: differences in CPS and TAP scores of positive and negative control cell lines stained with PD-L1 22C3, SP263 and 28-8 antibodies will also be compared. If sufficient data are available, PA and NA will be calculated and displayed alongside confusion matrices, providing information on whether the higher degree of staining of some of the IHC assays correspond to a higher PA or a lower NA. 4.3 *Clinical data collection*The following data will be collected at study entry, including but not limited to: • Demographic data: age and sex; • Basic relevant laboratory assessment, if available; • Primary disease information: date of diagnosis, primary tumour location (gastric versus gastro-oesophageal junction), histology, staging according to TNM (tumour–node–metastasis) based on American Joint Committee on Cancer latest recommendations; • Results of locally carried out tumour biomarker analyses, if available (e.g. HER2, microsatellite status, others according to local procedures, such as CLDN18.2 and FGFR2b); • Type of treatment received and setting, if available, including specifying if they included immunotherapy; and • Type of tumour specimen (from primary tumour versus metastatic sites; biopsy versus resection). Sites will be encouraged to prioritize biopsies from primary tumours as the predominant source of samples. However, samples from surgical resection sites and/or metastatic tumour biopsies will also be included to keep the study design pragmatic and real world. Minimally 30% of samples will be from primary tumour endoscopic biopsies and not more than 10% of samples from metastatic sites.
**Trial registration**	EORTC-RP-2321
**Ethics**	The study is conducted in adherence to the latest Declaration of Helsinki and Ethical Guidelines for Clinical Studies. The study protocol has received approval from the independent ethical review committee of EORTC and has been submitted to the local ethical committees. Before enrolment, written informed consent will be obtained from all patients. In specific sites, patients who have passed away may have sample access with a waiver of consent after local ethics approval.
**Value of the trial in progress**	• The ASPIRE study is the first global study evaluating the interchangeability of PD-L1 scoring systems and assays in GC. • The study will provide data to harmonize the quantification and qualification of PD-L1 expression in GC. • The results of the trial will help better select patients who derive benefit when treated with immunotherapy.

## Description of protocol

### Introduction

Immune checkpoint inhibitors (ICIs) targeting the programmed cell death protein 1 (PD-1)/programmed death-ligand 1 (PD-L1) pathway are now the standard of care for patients with various advanced and metastatic cancers, including gastro-oesophageal cancer (GC).^1^ Many studies have demonstrated that GCs with higher levels of PD-L1 expression tend to derive higher benefits from treatment with anti-PD-1 blockade.^2^ Thus, evaluation of PD-L1 expression via immunohistochemistry (IHC) has become an integral part of the treatment algorithm for patients with metastatic GC.

Various standardized IHC PD-L1 antibody assays (e.g. Dako 22C3, Dako 28-8, Ventana SP142 and Ventana SP263) were used in clinical trials, proving the efficacy of various ICIs.^3^ However, they have not been recommended as specific companion diagnostics, leading to confusion over the interchangeability and reproducibility of these assays.^1^^,^^4^ Each assay requires a different staining protocol, equipment and cut-offs, leading to a potential source of confusion among clinicians and pathologists, and a logistical hassle for laboratories and hospitals.

For example, in this context, CheckMate 649 was a randomized phase III trial that demonstrated the benefit of adding nivolumab to chemotherapy in the first-line treatment of metastatic gastric or oesophageal adenocarcinomas.^5^ The study analysed various PD-L1 combined positive score (CPS) subgroups as primary and secondary endpoints. While the study demonstrated benefit in the all-randomized CPS ≥1 and CPS ≥5 populations, there was significant controversy on the benefit in the PD-L1-low-expressing population (CPS < 5).^5^ This led to different regulatory approvals in various parts of the world, with the United States Food and Drug Administration (FDA) approving nivolumab regardless of CPS and the European Medicines Agency (EMA) approving nivolumab only for patients with a PD-L1 IHC score of CPS ≥5. The specific antibody used to score CPS in CheckMate 649 was the Dako 28-8 antibody. In contrast, several randomized phase III trials with pembrolizumab (anti-PD-1 antibody) have been designed only using the Dako 22C3 assay to select PD-L1-positive populations.^2^ This has led to the EMA approving pembrolizumab for these specific indications.^1^^,^^4^^,^^6^

Much uncertainty exists regarding the concordance of CPSs among different PD-L1 assays. Additionally, the recent FDA approval of tislelizumab (anti-PD-1) in combination with chemotherapy as the first-line treatment of patients with locally advanced unresectable or metastatic GC,^7^ based on the results of the phase III RATIONAL 305 trial,^8^ led to the use of the tumour area positivity (TAP) score for PD-L1 in clinical practice.

Thus, with an increasing number of approved ICIs and corresponding companion IHC assays,^2^ there is an unmet clinical need to demonstrate the concordance among these assays to allow interchangeable utilization in the clinic.^9^

Therefore, the assessment of PD-L1 and the methodology used are crucial to selecting patients as candidates for immunotherapy. However, no studies comparing the methods to assess PD-L1 positivity in GC (according to too many scoring systems, e.g. CPS, TAP) and the antibodies used for IHC are available.^10^^,^^11^

This study aims to compare different PD-L1 scoring systems and assays to simplify and/or harmonize the quantification and qualification of PD-L1 expression in the GC setting and ease the translation of endpoints in the companion diagnostic setting.

## Methods and analysis

### Study design

The ASPIRE trial is a stomach cancer PD-L1 biomarker European initiative conducted by the European Organisation for Research and Treatment of Cancer Gastrointestinal Tract Group (EORTC GITCG) and the National University Health System, Singapore, as an independent academic study. It aims to compare different PD-L1 scoring systems and assays to simplify and harmonize the quantification and qualification of PD-L1 expression in the GC setting and ease the translation of endpoints in the companion diagnostic setting.

One hundred and fifty formalin-fixed, paraffin-embedded (FFPE) primary tumour biopsy or resection samples will be collected through their routine clinical practice from European and Asian centres. The goal is to obtain global data on Asian and Western populations.

Biopsy or surgical specimen FFPE blocks will be sent centrally to the laboratory at the National University Health System, Singapore. Subsequently, a group of 10-15 international gastrointestinal (GI) experts and clinically active pathologists will score the samples for PD-L1 using the PD-L1 IHC 22C3 and 28-8 pharmDx assays, and the Ventana PD-L1 (SP263) assay. Ultimately, the correlation between different scoring systems and assays will be assessed (CPS and TAP).

**Figure 1:**
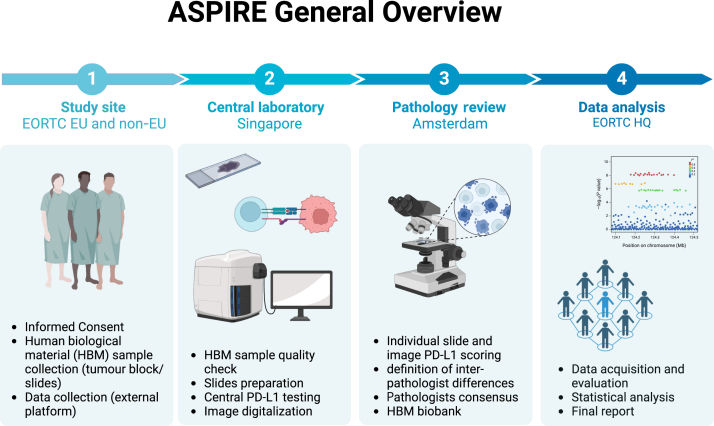
**ASPIRE study design general overview.** EORTC, European Organisation for Research and Treatment of Cancer; EU, European Union; HQ, headquarter; PD-L1, programmed death-ligand 1.

The study design general overview is summarized in Figure 1. Additional details are shown in Specifications Table.

### Patients

Patients with a histologically confirmed diagnosis of gastric or gastro-oesophageal junctional adenocarcinomas and availability of tumour samples from biopsy or resection obtained within the past 3 years are eligible for the analysis.

Written informed consent will be obtained from all patients before enrolment. In specific sites, patients who have passed away may have sample access with a waiver of consent after local ethics approval. The study is conducted in adherence to the latest Declaration of Helsinki and Ethical Guidelines for Clinical Studies. The study protocol has received approval from the independent ethical review committee of EORTC (study number: EORTC-RP-2321) and has been submitted to the local ethical committees.

### Endpoints

The primary objectives are to measure the interchangeability of the PD-L1 expression among IHC assays 22C3, SP263 and 28-8 and the comparison between the scoring systems CPS and TAP in the samples after dichotomization at 1 and 5 cut-offs.

The secondary endpoints are:

- the difference among the CPS and TAP scores in the samples stained with 22C3, SP263 and 28-8 antibodies;
- the concordance among PD-L1 expression status after dichotomizing of the TAP and CPS scores at other cut-offs than 1 and 5;
- the cut-off that would result in the smallest difference among IHC assays;
- the inter-pathologist variability, quantified by way of the intraclass correlation coefficient (ICC) of CPS and TAP scores; and
- the inter-pathologist variability, quantified using the Fleiss kappa (κ) statistic (FKS) on dichotomized scores at multiple cut-off levels.

The exploratory endpoints evaluate the co-expression of PD-L1 with other biomarkers of interest, including Epidermal growth factor receptor 2 (HER2), Claudin 18.2 (CLDN18.2) and fibroblastic growth factor receptor 2b (FGFR2b), and estimate the absolute performance among the IHC assays: differences in CPS and TAP scores of positive and negative control cell lines stained with 22C3, SP263 and 28-8 antibodies.

### Clinical data collection

The following data will be collected at study entry, including but not limited to:

• Demographic data: age and sex;
• Basic relevant laboratory assessment, if available;
• Primary disease information: date of diagnosis, primary tumour location (gastric versus gastro-oesophageal junction), histology, staging according to TNM (tumour–node–metastasis);
• Results of locally carried out tumour biomarker analyses, if available (e.g. HER2, microsatellite status, others according to local procedures, such as CLDN18.2 and FGFR2b);
• Type of treatment received and setting, if available, including specifying if it included immunotherapy; and
• Type of tumour specimen (from primary tumour versus metastatic sites; biopsy versus resection). Sites will be encouraged to prioritize biopsies from primary tumours as the predominant source of samples. However, samples from surgical resection sites and/or metastatic tumour biopsies will also be included to keep the study design pragmatic and real-world. Minimally 30% of samples will be from primary tumour endoscopic biopsies and not more than 10% of samples from metastatic sites.

### Molecular analysis

All tissue blocks will be sent centrally to the laboratory at the National University Health System, Singapore. Serial tissue sections (4 μm) prepared from the paraffin blocks will undergo haematoxylin-eosin (H&E) and PD-L1 immunostaining with the Agilent PD-L1 28-8 pharmDx assay, the Agilent PD-L1 22C3 pharmDx assay and the Ventana SP263 PD-L1 assay. The 28-8 and 22C3 pharmDx assays will be carried out both on a Dako Autostainer Link 48 system, while the SP263 assay will be carried out on the Ventana BenchMark Ultra autostainer, along with positive and negative controls according to the manufacturer’s instructions.

All PD-L1-stained slides and matching H&E-stained sections will be digitally scanned, and images will be uploaded. The evaluation will be performed on both glass slides and/or digital scoring. At least 30% of samples will have dual glass slides and digital scoring to evaluate the concordance between the two methodologies. Working from individually configured remote computer stations, 10-15 GI expert pathologists from different countries across several continents will evaluate the specimens with results blinded to each other.

PD-L1 expression will be evaluated in both tumour cells and immune cells. For tumour cells, positive PD-L1 staining is defined as partial and/or complete membranous staining at any intensity (cytoplasmic staining is excluded). Immune cells (tumour-associated lymphocytes and macrophages) with membranous and/or cytoplasmic staining of any intensity will be counted. The CPS will be calculated by dividing the number of PD-L1-stained cells (tumour and immune cells) by the total number of viable tumour cells, multiplied by 100. A minimum amount of 100 tumour cells should be present, and the maximum CPS value should be set to 100. In addition to the CPS scoring system, the TAP score will be assessed. The TAP score is determined on the slide by visually aggregating/estimating the area covered by PD-L1-positive tumour cells and tumour-associated immune cells relative to the total tumour area. For further details regarding determining the TAP score, see Liu et al.^12^

All pathologist participants are required to undergo training and consensus on the scoring of the PD-L1 methodology. PD-L1 CPS and TAP will be scored following clinically relevant cut-offs.

### Statistical analysis

The number of samples needed for this study was estimated based on a lower boundary of 80% overall rate of agreement (ORA), below which two assays are considered significantly different; a two-sided type I error constraint alpha of 0.015 accounting for the three comparisons (among the three assays); and a two-sided type II error constraint beta of 0.20. For the lower boundary of a one-sided 95% confidence interval (CI) around a true rounded-off ORA of 90% to remain above 80%, a sample size of *n* = 127 is needed. Taking a margin of 10% resulted in an estimated sample size of *n* = 140.

The binary primary endpoint will be assessed through three 2 × 2 contingency tables depicting the number of slides that agree (*n*++ and *n*−−) or do not agree (*n*+− and *n*−+) between two of the IHC assays. For each contingency table, the sum of all counts in the table (*n*) corresponds to the number of slides for which both assays have produced a valid PD-L1 expression status. The association between the qualitative results of two IHC assays will be presented as the ORA:

$$\text{ORA}=\frac{n+++n--}{n}$$

The ORA between two IHC assays will always be accompanied by the 95% CI calculated by Wilson.

The continuous secondary endpoint on PD-L1 expression will be assessed through the within-slide differences among the mean CPS and TAP scores of the IHC assays and the statistical significance thereof. With three assays, there are three such inter-assay differences that will be calculated.

The concordance among PD-L1 expression status after dichotomizing of the TAP and CPS scores at other cut-offs than 1 and 5 will be calculated as mentioned above.

The best cut-off, i.e. the cut-off that results in the smallest difference among IHC assays, will be established as the cut-off that maximizes the sum of the positive (PA) and negative agreement (NA), where PA and NA are the symmetric counterparts of the positive percent agreement (PPA) and negative percent agreement (NPA) as defined by the FDA.

The inter-pathologist variability will be quantified by way of the ICC when dealing with continuous CPS and TAP scores and with the FKS when dealing with dichotomized PD-L1 statuses. For each combination of scoring system (TAP and CPS) and IHC assay (22C3, SP263 and 28-8), the continuous or dichotomized scores will be summarized allowing to assess whether the pathologist bias is comparable among scoring systems and IHC assays. The relation between the cut-off value and the amount of inter-pathologist variation will be demonstrated for the dichotomized scores.

The co-expression of PD-L1 with other biomarkers of interest, including HER2, CLDN18.2 and FGFR2b, will be tabulated. Co-expression will be tested using multiple IHC with a panel of various markers, including, but not limited to, CD45, CD8, CD4, CD68, FGFR, CLDN18.2, CEACAM5, TROP2, HER2, EBER, PanCK and SMA.

To estimate the feasibility of measuring the absolute performance among the IHC assays, differences in CPS and TAP scores of positive and negative control cell lines stained with 22C3, SP263 and 28-8 antibodies will also be compared. If sufficient data are available, PPA and NPA will be calculated and displayed alongside confusion matrices, providing information on whether the higher degree of staining of some of the IHC assays corresponds to a higher PPA or a lower NPA.

## Results dissemination policy

The study’s results will be presented at the main international congresses in the field and published in a peer-reviewed international oncology journal.

## Conclusions

In conclusion, the ASPIRE study represents a foundational initiative in the global landscape of immune-oncology translational GC research, aiming to address the critical need for standardization and harmonization in assessing PD-L1 expression for immunotherapy selection. By evaluating the interchangeability of PD-L1 scoring systems and assays, ASPIRE aims to streamline the quantification and qualification of PD-L1 expression, facilitating more consistent and accurate patient stratification. Through multiple comparison and correlation analyses involving an international panel of experts and geographically and biologically varied patient samples, the ASPIRE study promises to provide valuable insights that could significantly impact clinical practice, ultimately optimizing patient care and treatment outcomes.
